# Colonization by the Mycorrhizal Helper *Bacillus pumilus* HR10 Is Enhanced During the Establishment of Ectomycorrhizal Symbiosis Between *Hymenochaete* sp. Rl and *Pinus thunbergii*

**DOI:** 10.3389/fmicb.2022.818912

**Published:** 2022-03-07

**Authors:** Ya-Hui Wang, Wei-Liang Kong, Mei-Ling Zhu, Yun Dai, Xiao-Qin Wu

**Affiliations:** ^1^Co-Innovation Center for Sustainable Forestry in Southern China, College of Forestry, Nanjing Forestry University, Nanjing, China; ^2^Jiangsu Key Laboratory for Prevention and Management of Invasive Species, Nanjing Forestry University, Nanjing, China

**Keywords:** mycorrhizal helper bacteria, ectomycorrhizal fungi, mycorrhizosphere, colonization, biofilm, motility

## Abstract

There are complex interactions between mycorrhizal helper bacteria (MHBs) and ectomycorrhizal (ECM) fungi, with MHBs promoting mycorrhizal synthesis and ECM fungi regulating plant rhizobacterial colonization, diversity, and function. In this study, to investigate whether the ECM fungus *Hymenochaete* sp. Rl affects the survival and colonization of the MHB strain *Bacillus pumilus* HR10 in the rhizosphere, the biomass of *B. pumilus* HR10 was measured in the rhizosphere and mycorrhizosphere. In addition, extracts of *Hymenochaete* sp. Rl and *Pinus thunbergii* were evaluated for their effect on *B. pumilus* HR10 colonization (growth, sporulation, biofilm formation, extracellular polysaccharide and extracellular protein contents, flagellar motility, and expression of colonization-related genes). The results showed that inoculation of *Hymenochaete* sp. Rl significantly increased the biomass of *B. pumilus* HR10 in the rhizosphere; however, while extracts of *Hymenochaete* sp. Rl and *P. thunbergii* did not affect the biomass or spore formation of HR10, they did affect its biofilm formation, extracellular polysaccharide and extracellular protein production, and flagellar motility. Furthermore, the addition of symbiont extracts affected the expression of chemotaxis-related genes in HR10. When the extracts were added separately, the expression of *srf* genes in HR10 increased; when the extracts were added simultaneously, the expression of the flagellin gene *fliG* in HR10 increased, but there was no significant effect on the expression of srf genes, consistent with the results on biofilm production. Thus, *Hymenochaete* sp. Rl and *P. thunbergii* roots had a positive effect on colonization by *B. pumilus* HR10 at the rhizosphere level through their secretions.

## Introduction

Ectomycorrhizal (ECM) fungi establish symbiosis with the roots of most trees in boreal and temperate ecosystems and are major drivers of nutrient circulation between trees and the soil (Plassard and Dell, 2010; Martin et al., 2016; Moreau et al., 2019; Tedersoo et al., 2020). Thus, ECM fungi enhance the ability of trees to absorb various mineral nutrients from the soil, and their hyphae are sometimes considered extensions of the root system (Dietz et al., 2011; Martin et al., 2016; Oldroyd and Leyser, 2020). As research has progressed, mycorrhizae have been found to be a complex microecological system (Guo et al., 2016). Mycorrhizal fungi-plant symbionts and rhizosphere fungi, bacteria, actinomycetes, and other microorganisms are found in close interaction patterns in physical structures, active ingredient metabolism, and functional exertion (Morgado et al., 2015; Santalahti et al., 2018; Li et al., 2019; Steidinger et al., 2019). On the one hand, mycorrhizal fungi interact with the soil bacterial and modify the rhizosphere microbial community. This part of the study is mostly seen in the effect of inoculation of arbuscular mycorrhizal (AM) fungi on the growth of rhizosphere bacteria, and the effect of ECM fungi on rhizosphere bacteria is rarely reported. Studies have demonstrated that colonization by mycorrhizal fungi decreases (Christensen and Jakobsen, 1993; Wamberg et al., 2003; Cavagnaro et al., 2006) but also increases (Aarle et al., 2003; Albertsen et al., 2006) or have no effect (Olsson et al., 1996) on the microbial biomass due to differences in plant species, experimental duration, root growth activity, exudate composition and/or amount, and carbohydrate metabolism of the plant. On the other hand, rhizosphere bacteria can affect the growth and colonization of mycorrhizal fungi (Mechri et al., 2014). Labbe et al. (2014) analyzed the effects of 23 individual *Pseudomonas* strains the growth and colonization of *Laccaria bicolor*. Nineteen of the 23 *Pseudomonas* strains promoted the growth of *L. bicolor*, three of them had positive effects on mycorrhizal formation and one strain inhibited mycorrhization; two strains significantly inhibited the growth of *L. bicolor* and inhibited mycorrhization. These bacterial strains that positively influenced the establishment and functioning of mycorrhizal symbioses were categorized as mycorrhizal helper bacteria (MHBs; Garbaye, 1994). Researchers have screened for MHBs that promote mycorrhizal formation in plants from a variety of different habitats. Current theory holds that MHB play a role in promoting the mycelial growth of mycorrhizal fungi, reducing the concentration of toxic substances in the soil, promoting the development of host plant roots, and increasing mycorrhizal infection, thereby achieving the ultimate goal of improving the efficiency of mycorrhizal formation (Poole et al., 2001; Vivas et al., 2006; Deveau et al., 2007; Zhao et al., 2013; Armada et al., 2016; Shinde et al., 2019).

Studies have shown that MHBs have a benign interaction with mycorrhizal fungi, which is reflected not only in the promotion of EMC fungal growth and morphology by MHBs but also in the positive effect of EMC fungi on the biomass and colonization of MHBs (Frey-Klett et al., 2005; Deveau et al., 2010; Marupakula et al., 2016; Velez et al., 2018). During mycorrhization, the proliferation of MHBs in the rhizosphere prior to symbiosis can improve the receptivity of the roots to mycorrhizal formation (Aspray et al., 2006). Proliferating MHBs guarantee the supply of growth-promoting substances, which may also promote the growth of the fungus in its saprotrophic state in the soil or at the root surface, triggering or accelerating the germination of fungal propagules in soil (Nazir et al., 2010). Frey-Klett et al. (1997) showed that the survival of the MHB strain *Pseudomonas fluorescens* BBc6R8 in soil was significantly improved by the presence of the ECM strain *Laccaria bicolor* S238N; however, the biomass of *P. fluorescens* BBc6R8 was found to significantly decrease in the nonmycorrhizal Douglas-fir in the presence of *L. bicolor* S238N, indicating that this bacterial strain is more dependent on the presence of fungi than on the presence of roots. Additionally, under pure culture conditions, *P. fluorescens* BBc6R8 adheres to the surface of the mycelium of *L. bicolor* S238N, forming a biofilm-like structure (Frey-Klett et al., 2007). *Glomus mosseae*, an AM fungus, has improved the long-term survival of *P. fluorescens* 92rk, an MHB strain, in the rhizosphere of tomato plants (Gamalero et al., 2004, 2005).

Studies have shown that the interaction between MHBs and mycorrhizal fungi is mutualistic, and we isolated an MHB strain, *Bacillus pumilus* HR10, from the *Pinus thunbergii*–*Rhizopogon luteous* mycorrhizosphere, which can significantly promote the mycorrhizae and growth of *P. thunbergii* (Sheng et al., 2014; Wang et al., 2021), but the effect of the mycorrhizae on the rhizosphere of *B. pumilus* HR10 is not yet clear. Therefore, studies on the effects of mycorrhizae on *B. pumilus* HR10 rhizosphere colonization and biofilm formation are particularly important for understanding the interaction between mycorrhizal fungi and MHBs to promote pine growth, this not only supports the interaction between MHBs and mycorrhizal fungi, but also provides a basis for the application of the *B. pumilus* HR10 strain in the field. In these experiment, the biomass of *B. pumilus* HR10 in the mycorrhizosphere was detected by the plate counting method, and the growth, biofilm formation, extracellular polysaccharide and extracellular protein production ability, and flagellar motility of *B. pumilus* HR10 after treatment with *Hymenochaete* sp. Rl mycelium and *P. thunbergii* root extracts were investigated to understand the effect of mycorrhizae on the rhizosphere of *B. pumilus* HR10, and provide a theoretical basis for further utilization and development of *B. pumilus* HR10 to increase pine growth.

## Materials and Methods

### Microbial Strains and Culture Conditions

*Bacillus pumilus* HR10 was originally isolated from the rhizosphere soil of mycorrhizal seedlings of *Pinus thunbergii* (Sheng et al., 2014); it is a mycorrhizal helper bacterium that promotes the formation of *P. thunbergii*—*Hymenochaete* sp. Rl mycorrhizal symbiosis. *Bacillus pumilus* HR10 can also secrete antagonistic proteins for effective control of *Sphaeropsis* shoot blight (Dai et al., 2021) and can control pine seedling damping-off disease caused by *Rhizoctonia solani* because of its efficient colonization capacity (Zhu et al., 2020). It was maintained at −80°C in Luria-Bertani (LB; 10 g L^−1^ tryptone, 5 g L^−1^ yeast extract, and 5 g L^−1^ NaCl) medium with 25% glycerol. It was first grown on LB agar plates overnight at 30°C. Then, individual colonies were collected from culture plates to inoculate 25 ml of liquid LB medium, followed by incubation at 28°C and 200 rpm until an OD_600nm_ value of 1.0 was reached before their use for growth, spore formation, biofilm formation, and flagellar motility assays. *Hymenochaete* sp. Rl as the supplied experimental ectomycorrhizal fungus was originally isolated from the Zixi Mountain Forest Park, Yunnan Province, China (Yang, 2004). Fungal cultures were maintained on ZPD medium (boiled juice of 200 g L^−1^ potato, 20.0 g L^−1^ glucose, 2.5 g L^−1^ K_2_HPO_4_, 1.5 g L^−1^ MgSO_4_·7H_2_O, 0.05 g L^−1^ vitamin B_1_, and 15.0 g L^−1^ agar added to solid medium).

### Laboratory Conditions for Colonization Community Interactions

Two community conditions were considered in this experiment: pine with *B. pumilus* HR10; and pine with both *B. pumilus* HR10 and *Hymenochaete* sp. Rl. Pine seedlings were grown in tissue culture bottles containing soil, sand, and vermiculite (2:1:1). The mixture was crushed, passed through a 2 mm sieve, and autoclaved at 121°C for 90 min to eliminate the native microflora. The seeds were germinated by soaking in water for 24 h, and sterilized in 30% H_2_O_2_ for 30 min. Then these seeds were sown on water agar plates and incubated at 25°C in a greenhouse.

*Hymenochaete* sp. Rl was the first community member introduced into the bottle communities. The fungus was grown on liquid ZPD medium at 25°C for approximately 3 weeks with shaking at 150 rpm. The fungus was collected and washed three times with sterile water, and the hyphae were cut into small hyphal segments with a tissue crusher. The seedlings were transferred to bottles each containing 100 g of soil, which were introduced with 0.05 g of mycorrhizal inoculum. For nonmycorrhizal conditions, an equivalent volume of sterile water was added instead of fungal slurry. Around 10 days after the pine seedlings were planted, 5 ml bacterial suspensions [10^7^ colony forming units (cfu)/g soil] were slowly poured into each bottle around the stem of the seedling. The *B. pumilus* HR10 bacterial culture inoculum was grown in 20 ml of LB medium overnight at 28°C with shaking at 200 rpm. After harvesting by centrifugation at 1,500 *g* for 10 min, the sediments were washed and resuspended in sterile water to obtain an approximate bacterial number of 5 × 10^8^ cfu per ml. All seedlings were grown in greenhouse at 25°C (12-h light, 12-h dark time). The seedlings were transplanted after the cotyledons developed. The experiment was performed with three biological replicates per experimental condition for a total of six bottles that were sealed with sealing film to ensure pure cultures. The communities were allowed to grow for an additional 3 months after the addition of bacteria and then harvested to measure the biomass of *B. pumilus* HR10 in the rhizosphere soil and the pine root surfaces.

### Biomass of *Bacillus pumilus* HR10 Assay

The roots were rinsed with sterile water to remove surface soil and then observed by scanning electron microscopy. In addition, 0.1 g of rhizosphere soil was placed in a shake flask containing 10 ml of sterile water. After shaking at 200 rpm for 10 min at 30°C, the mixture was diluted by the appropriate amount and coated in solid LB medium. After 24 h, the number of colonies was recorded; the number of colonies was used to estimate the extent of colonization. The root surface biomass was measured by replacing 0.1 g of rhizosphere soil with a sample of the root system (10 cm in length) after gentle rinsing with sterile water in a shake flask.

### Growth and Spore Formation of *Bacillus pumilus* HR10 *in vitro*

Preparation of the *Hymenochaete* sp. Rl mycelium and *P. thunbergii* root extracts: A certain amount of *Hymenochaete* sp. Rl mycelium and *P. thunbergii* roots were ground into a powder in liquid nitrogen, 10 volumes (w/v) of PBS buffer were added, and the samples were sterilized with a 0.22 μm membrane filter. Then, the *Hymenochaete* sp. Rl mycelium and *P. thunbergii* root extracts were obtained. Cultures of *B. pumilus* HR10 were grown to an OD_600nm_ value of 1.0 and diluted 100-fold in LB medium supplemented with an extract of *Hymenochaete* sp. Rl mycelium and/or *P. thunbergii* roots, and then shaken at 30°C and 200 rpm. Samples taken every 6 h were diluted by an appropriate multiple and coated in LB solid medium. After 24 h, the number of colonies was recorded. Continuous measurements over 120 h formed a growth curve of *B. pumilus* HR10. Sampling for the spore formation curve of *B. pumilus* HR10 was identical to the growth curve except that the samples were warmed at 85°C for 10 min before dilution.

### Biofilm Formation of *Bacillus pumilus* HR10

After culturing overnight, the *B. pumilus* HR10 dilution was coated in LB solid medium supplemented with the above extract of *Hymenochaete* sp. Rl mycelium and/or *P. thunbergii* roots, and the colony morphology was observed by Zeiss microscopy after standing at 28°C for 24 h. Biofilm formation was measured using the microplate assay. Briefly, cultures of *B. pumilus* HR10 were grown to an OD_600nm_ value of 1.0, and then diluted 100-fold in LB medium supplemented with an extract of *Hymenochaete* sp. Rl mycelium or/and *P. thunbergii* roots. The diluted culture was dispensed into a 96-well polypropylene microtiter plate with 200 μl per well. After incubation at 30°C for 48 h, the culture medium was removed, and unattached cells were washed off by rinsing each well with 250 μl of 10 mM PBS (pH 7.2). A total of three washes were performed. Subsequently, 2 ml of 0.1% (v/v) crystal violet solution was added to each well and the plate was incubated at room temperature for 30 min and rinsed three times with deionized water (250 μl per rinse). Crystal violet was dissolved by the addition of 250 μl ethanol. The absorption of the eluted stain was measured at a wavelength of 590 nm.

Overnight cultures of *B. pumilus* HR10 were inoculated into LB medium (0.1%, v/v) supplemented with extracts of *Hymenochaete* sp. Rl mycelium and/or *P. thunbergii* roots, and the mixture was shaken at 200 rpm and 30°C to an OD_600_ ≈ 1.5. The culture was allowed to stand for 48 h. The OD_595_ was measured by a microplate reader, and then the samples were washed three times with PBS buffer to remove floating bacteria. Then, 1 ml of a 0.01 M KCl solution was added and mixed to obtain suspensions. These suspensions were sonicated (5 s each time, 5 s gap, 5 cycles). The samples were centrifuged at 4,000 *g* for 20 min at 4°C, and sterilized by a 0.22 μm membrane filter. Then 200 μl of concentrated sulfuric acid was added to 100 μl of the above filtrate, and after standing for 30 min, 25 μl of phenol solution (6%) was added. The OD_490_ was determined after incubating at 90°C for 5 min, and the relative content of the extracellular polysaccharide was determined based on the OD_490_/OD_595_ value. Then, 200 μl of forinol reagent solution was added to 40 μl of the above filtrate and after standing for 10 min at room temperature, 20 μl of forinol reagent B was added, followed by a 30 min incubation at room temperature. Finally, the OD_750_ was determined, and the relative content of the extracellular protein was calculated from the OD_490_/OD_595_ value.

### Swarming and Swimming Motility Assay

To measure the swarming motility of *B. pumilus* HR10, motility agar (5 g L^−1^ agar, 10 g L^−1^ tryptone, and 5 g L^−1^ NaCl) was used. Swimming motility assays were performed on 0.5% (w/v) agar LB plates supplemented with 0.5% (w/v) glucose (Hou et al., 2019; Zhu et al., 2019). After solidification, plates were briefly dried at room temperature and spot inoculated with 2 μl aliquots taken directly from overnight LB cultures, and these plates were incubated face up at 28°C for 16 h. Experiments were repeated in triplicate and the data are presented as averages over three replicate plates.

### Real-Time PCR Assay

*Bacillus pumilus* HR10 was cultured on LB agar plates containing an extract of *Hymenochaete* sp. Rl mycelium or/and *P. thunbergii* roots. After 12 h, the cells were harvested and subjected to total RNA extraction using TRIzol (Invitrogen) according to the manufacturer’s instructions. Approximately 1 μg of total RNA was reverse transcribed into cDNA using a HiScript II Q RT SuperMix for qPCR Kit (R223-01, Vazyme, China). The reaction mixture consisted of 2 μl template cDNA, 12.5 μl ChamQ™ SYBR qPCR Master Mix (Q311-02, Vazyme, China), 0.5 μl each of the forward and reverse primers (10 mM; Table 1), and 9.5 μl RNA free water. Amplification was performed with a Step-One Thermal Cycler (Applied Biosystems 7500, United States) and consisted of 40 cycles of denaturation at 95°C for 15 s, annealing at 95°C for 10 s, and extension at 60°C for 43 s. The *gyrB* gene was used as housekeeping control (Zhao et al., 2011). The result was analyzed by the 2^-ΔΔ^CT method.

**Table 1:** Primer sequences used in this study.

Gene name	Forward primer sequences	Reverse primer sequences
*gyrB*	GAGGGAGTCGGTAATGGTTCTT	CGAAGCTGGCTTTAAAACCG
*sfp*	GAGAATATCACCGGAATTGAAAA	GCTTTCCTTCCAGCCATAGC
*fliG*	TACCCAAACGGGCGGAGTC	CGACCATACGCTGCGACA
*CheR*:	CAAGTTTCTCCTAAGCCGTTCA	TAGCCAGCGATGCCGTAA

### Statistical Analysis

Three replicate trials were carried out for each sample, and all the experiments were repeated three times. Data were analyzed by one-way ANOVA using DPS 9.50 software, and expressed as the means ± SDs. Statistical significance was considered at the *p* < 0.05 level. All graphs were drawn with Excel 2010 software.

## Results

### The Biomass of *Bacillus pumilus* HR10 Was Improved in a *Pinus thunbergii*-*Hymenochaete* sp. Rl Mycorrhizal Rhizosphere

**Figure 1:**
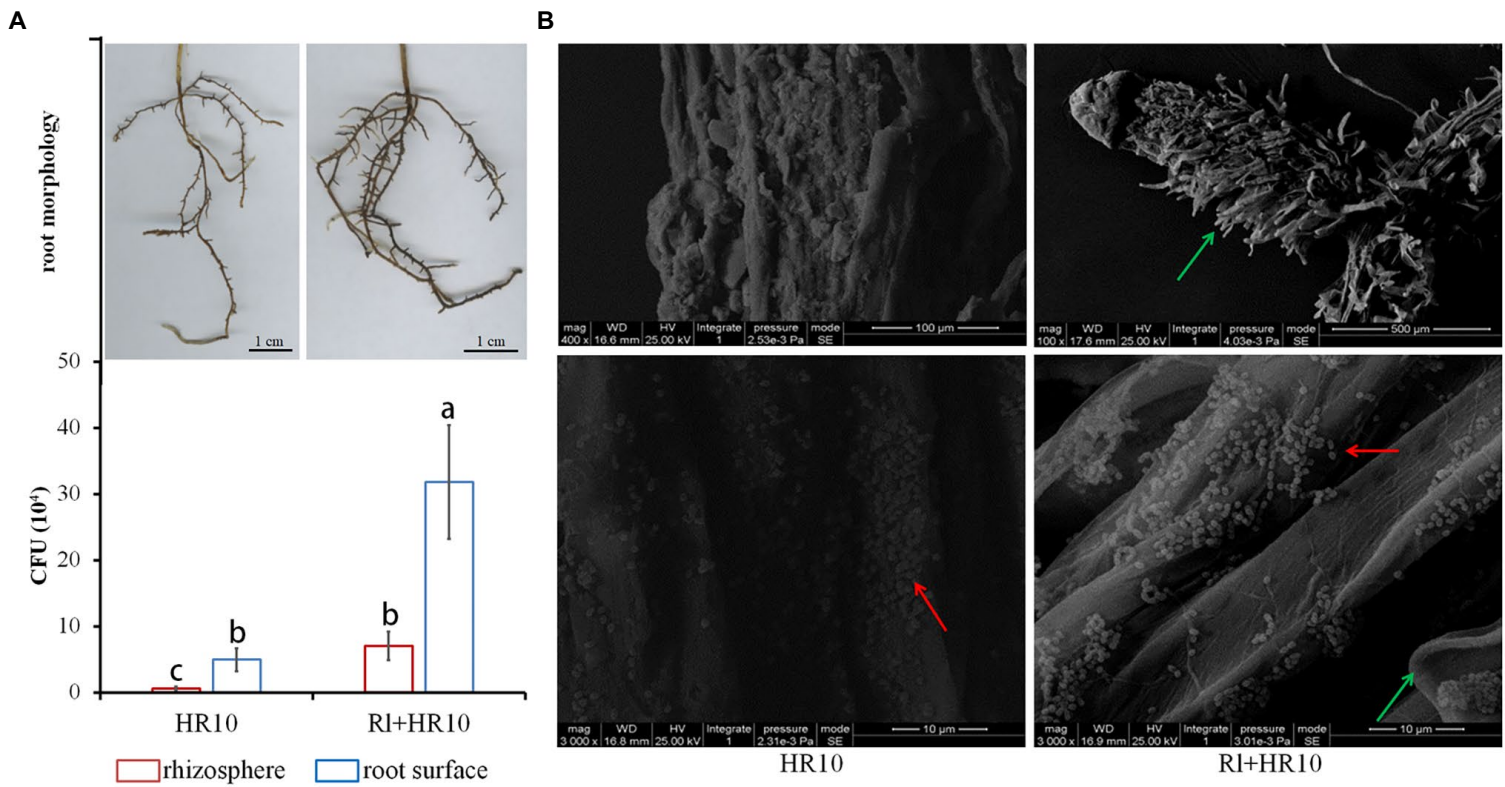
The biomass of *Bacillus pumilus* HR10. **(A)** The root of *Pinus thunbergii* (top) and statistics for the number of *B. pumilus* HR10 colonies in the rhizosphere soil and root surface of pine seedlings (bottom). **(B)** *Bacillus pumilus* HR10 on the root surface of pine seedlings was observed by scanning electron microscopy. HR10 indicates the pine inoculated with the MHB *B. pumilus* HR10. Rl + HR10 indicates the pine inoculated with both *Hymenochaete* sp. Rl and *B. pumilus* HR10. The red arrow points to HR10, and the green arrow points to the hyphae of *Hymenochaete* sp. Rl. Different letters above the chart columns indicate significant differences among treatments (*P* ≤ 0.05).

Sterile 1-month-old black pine seedlings were inoculated with *Hymenochaete* sp. Rl or/and *B. pumilus* HR10. After 3 months, the root surface was observed by scanning electron microscopy, and the rhizosphere soil and pine roots were taken to measure the biomass of *B. pumilus* HR10. The results showed (Figure 1) that the number of *B. pumilus* HR10 colonies on the root surface of the pines was higher than that in the rhizosphere soil, indicating that *B. pumilus* HR10 interacts with the roots of pine to increase its biomass and colonize the pine root surface. The biomass of *B. pumilus* HR10 in the rhizosphere of pines was affected by *Hymenochaete* sp. Rl, with the *B. pumilus* HR10 colony number increasing significantly in both the rhizosphere soil and the root surface of the pines inoculated with *Hymenochaete* sp. Rl; therefore, the mycorrhizal symbiosis formed by *Hymenochaete* sp. Rl-infected *P. thunbergii* can also improve *B. pumilus* HR10 survivability in the rhizosphere soil. The same result was observed by scanning electron microscopy.

### Extracts of *Pinus thunbergii* and *Hymenochaete* sp. Rl Had No Effect on the Growth or Spore Formation of *Bacillus pumilus* HR10

**Figure 2:**
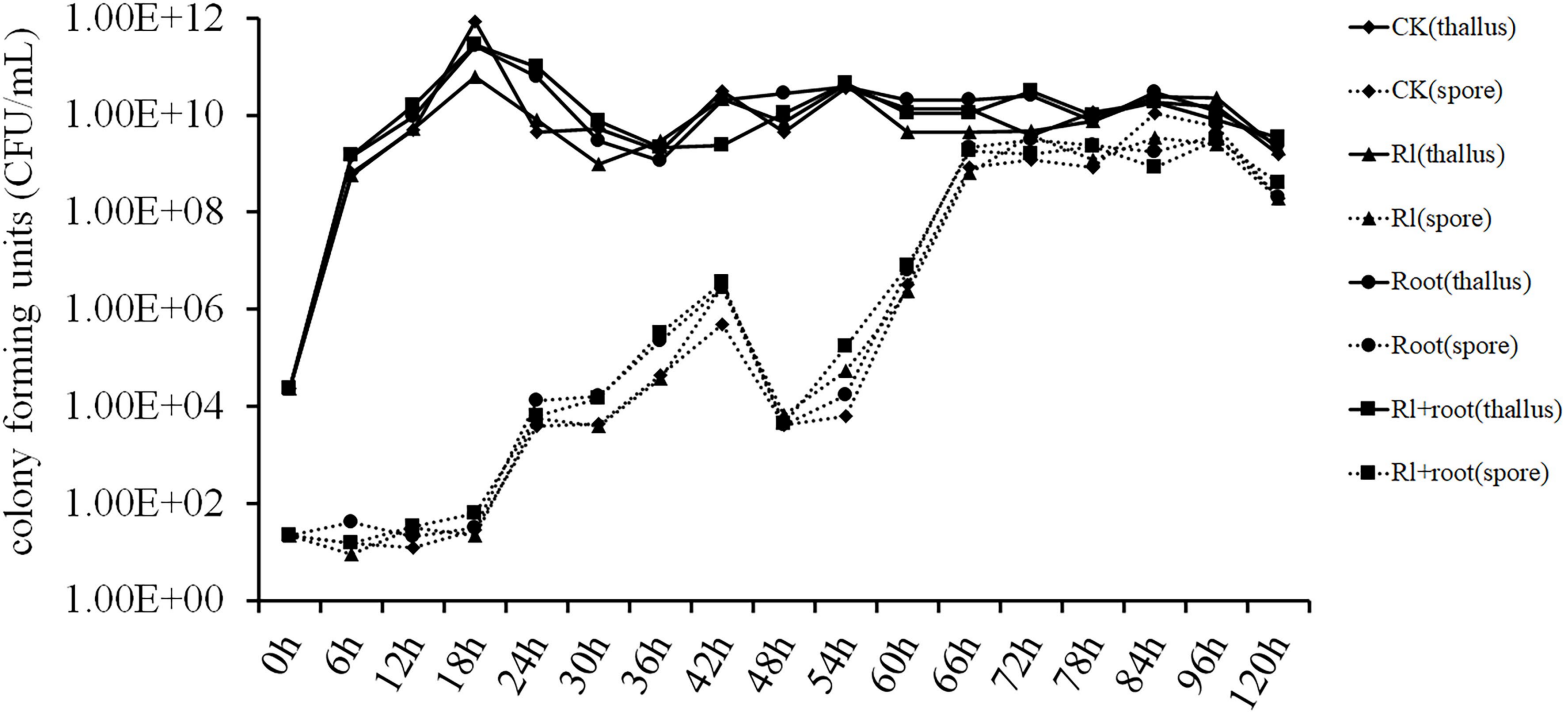
The effect of *Pinus thunbergii* and *Hymenochaete* sp. Rl on the growth and spore formation of *Bacillus pumilus* HR10. CK indicates when the medium was supplemented with 1xPBS buffer. Rl indicates when the medium was supplemented with the extract of *Hymenochaete* sp. Rl mycelium. Root indicates when the medium was supplemented with the extract of *P. thunbergii* roots. Rl + root indicates when the medium was supplemented with both *Hymenochaete* sp. Rl mycelium and *P. thunbergii* root extracts.

Luria-Bertani medium was added to the filter-sterilized extracts of *Hymenochaete* sp. Rl mycelium or/and the pine roots. *Bacillus pumilus* HR10 was inoculated in different treatment media, and then the number of *B. pumilus* HR10 thalli and spores in the different treatment media were measured. The statistical results showed that the extracts of *P. thunbergii* and *Hymenochaete* sp. Rl had no significant effect on the biomass or spore formation of *B. pumilus* HR10 under the experimental conditions tested (Figure 2).

### Biofilm Formation of *Bacillus pumilus* HR10 Was Affected by Extracts of *Pinus thunbergii* and *Hymenochaete* sp. Rl

**Figure 3:**
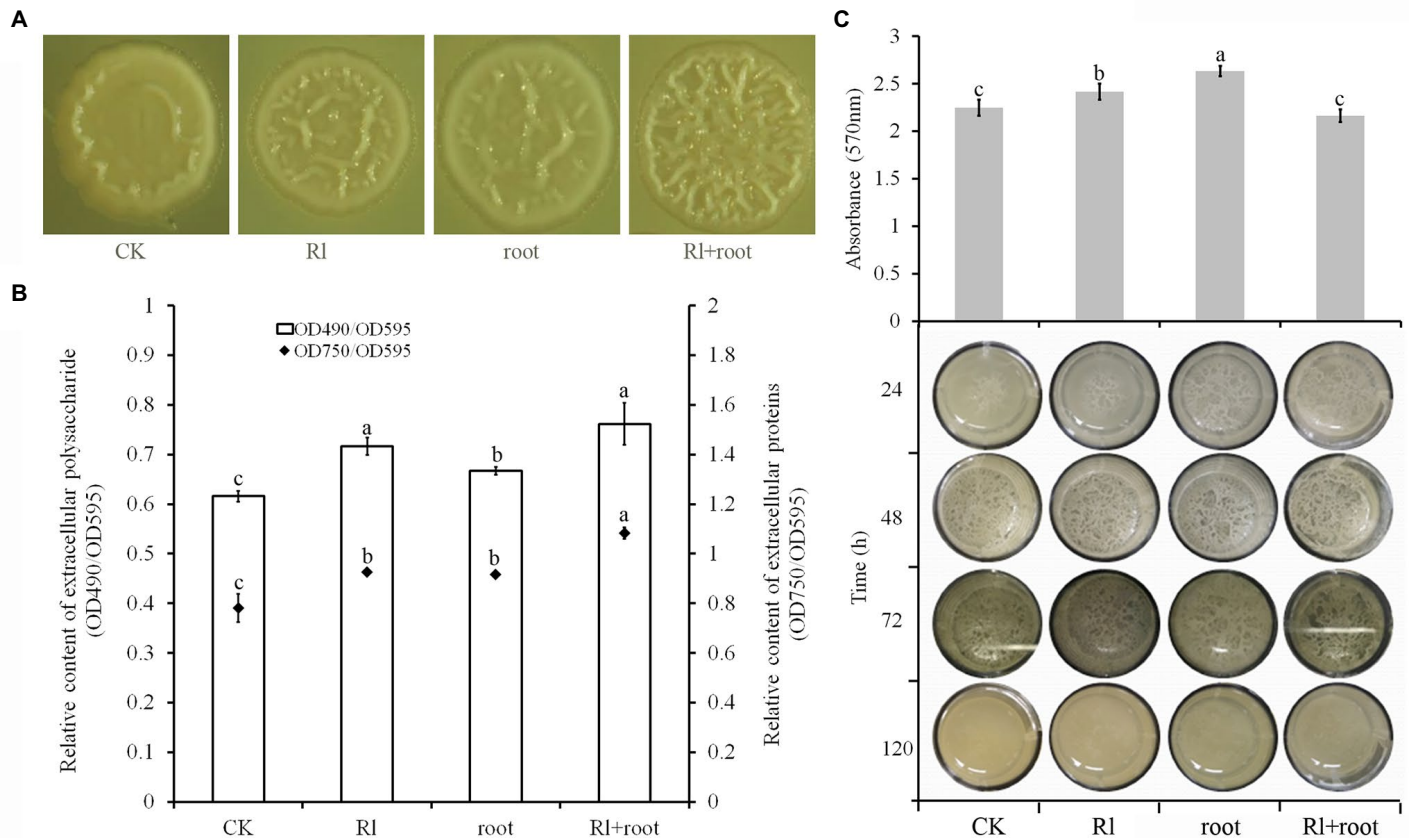
The effect of *Pinus thunbergii* and *Hymenochaete* sp. Rl on *Bacillus pumilus* HR10 biofilm formation. **(A)** Colony morphology of *B. pumilus* HR10. **(B)** Relative content of extracellular polysaccharides and extracellular proteins in the biofilms of *B. pumilus* HR10. **(C)** *Bacillus pumilus* HR10 biofilm formation and degradation (bottom), as well as *B. pumilus* HR10 biofilm quantification by crystal violet after 48 h (top). CK indicates medium supplemented with 1xPBS buffer. Rl indicates medium supplemented with *Hymenochaete* sp. Rl mycelium extract. Root indicates medium supplemented with *P. thunbergii* root extracts. Rl + root indicates medium supplemented with both *Hymenochaete* sp. Rl mycelium and *P. thunbergii* root extracts. Different letters above the chart columns indicate significant differences among treatments (*P* ≤ 0.05).

Microscopic observation of the colony morphology of *B. pumilus* HR10 showed that supplementation of the media with both *Hymenochaete* sp. Rl mycelium and *P. thunbergii* root extracts significantly increased the number of wrinkles on the surface of the colonies. Followed by the treatments with extracts of *Hymenochaete* sp. Rl mycelium or *P. thunbergii* roots, the colonies treated with only 1xPBS had the fewest folds on their surfaces (Figure 3A). The production of extracellular polysaccharide and extracellular protein by *B. pumilus* HR10 was significantly enhanced by adding *Hymenochaete* sp. Rl mycelium or/and *P. thunbergii* root extracts. *Bacillus pumilus* HR10 extracellular polysaccharide and extracellular protein were the most abundant when both *Hymenochaete* sp. Rl mycelium and *P. thunbergii* root extracts were added to the medium, followed by treatment with *Hymenochaete* sp. Rl mycelium extract alone (Figure 3B). *Bacillus pumilus* HR10 was cultured to the logarithmic growth phase in LB medium supplemented with *Hymenochaete* sp. Rl mycelium and/or *P. thunbergii* root extracts, and then 2 ml was transferred to 24-well plates and the plates were statically cultured. It was observed that the addition of *P. thunbergii* root extract and the combined extract of *Hymenochaete* sp. Rl mycelium and *P. thunbergii* root advanced the biofilm formation time of *B. pumilus* HR10, and the addition of *P. thunbergii* root delayed the biofilm degradation time (Figure 3C bottom). Biofilms formed following static incubation for 48 h were stained with crystal violet, and we found that there were significantly more biofilms formed when *Hymenochaete* sp. Rl mycelium or *P. thunbergii* root extracts were added (Figure 3C top).

### The Motility of *Bacillus pumilus* HR10 Was Affected by Extracts of *Pinus thunbergii* and *Hymenochaete* sp. Rl

**Figure 4:**
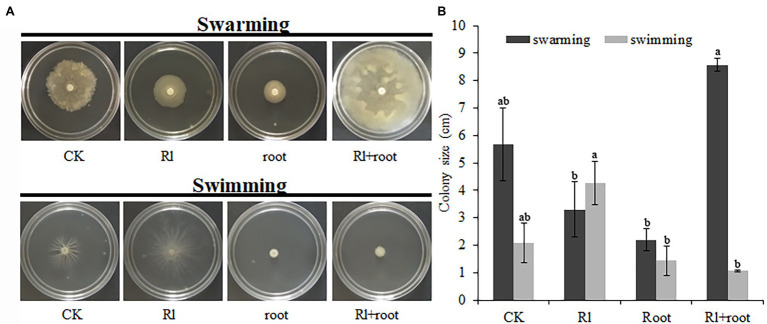
The effect of *Pinus thunbergii* and *Hymenochaete* sp. Rl on the motility of *Bacillus pumilus* HR10. **(A)** The swarming and swimming of *B. pumilus* HR10. **(B)** *Bacillus pumilus* HR10 colony size on plates used for swarming and swimming detection. Different letters above the chart columns indicate significant differences among treatments (*P* ≤ 0.05).

The swarming of *B. pumilus* HR10 was significantly enhanced by the extract of *Hymenochaete* sp. Rl mycelium. The combined treatment of extracts of *Hymenochaete* sp. Rl mycelium and *P. thunbergii* roots resulted in the strongest *B. pumilus* HR10 swimming activity. *Bacillus pumilus* HR10 swimming was significantly inhibited by the *P. thunbergii* root extracts alone (Figure 4A). The *B. pumilus* HR10 colony size was minimal in the swarming detection medium when the extracts of *P. thunbergii* roots or both extracts of *Hymenochaete* sp. Rl mycelium and *P. thunbergii* roots were added (Figure 4B).

### Extracts of *Pinus thunbergii* and *Hymenochaete* sp. Rl Affected the Expression of Genes Related to Colonization in *Bacillus pumilus* HR10

**Figure 5:**
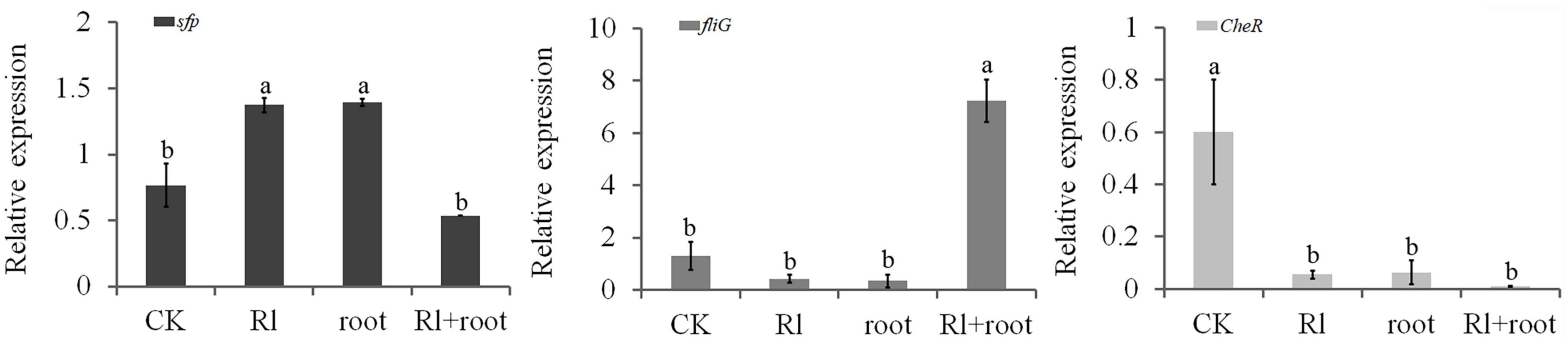
Effect of *Pinus thunbergii* roots and the ectomycorrhizal fungus *Hymenochaete* sp. Rl on colonization-related gene expression of *Bacillus pumilus* HR10. Different letters above the chart columns indicate significant differences among treatments (*P* ≤ 0.05).

The assay showed that the expression of the *sfp* gene was increased by the *Hymenochaete* sp. Rl mycelium extract and by *P. thunbergii* root extract; the *sfp* gene is involved in the synthesis of surfactins that play an important role in the formation of biofilms. Expression of the *fliG* gene, which regulates flagellar motility, was also increased by treatment with both extracts. The gene *CheR* encodes the chemotaxis receptor that catalyzes methyl-accepting chemotaxis protein (MCP) methylation in the flagellar motility system; its expression was downregulated by the addition of *Hymenochaete* sp. Rl mycelium or/and *P. thunbergii* root extracts (Figure 5). This finding suggests that the secretions of the ectomycorrhizal fungus *Hymenochaete* sp. Rl or pine roots may play an important role in the colonization of the rhizosphere by *B. pumilus* HR10.

## Discussion

Mycorrhizal helper bacteria are plant growth-promoting rhizosphere microorganisms that have a positive interaction with mycorrhizal fungi and host plants (Garbaye, 1994; Frey-Klett et al., 2007). MHBs can promote mycorrhizal mycelium growth (Schrey et al., 2005; Deveau et al., 2007, 2010; Zhao et al., 2013), increase host plant root development (Deveau et al., 2007; Labbe et al., 2014), increase opportunities for mycorrhizal fungi to come into contact host root systems, and influence mutual recognition between mycorrhizal fungi and host plants (Schrey et al., 2005). In addition, mycorrhizal fungi and host plants not only provide habitats for MHBs but also provide rich carbon sources, such as trehalose (Duponnois and Kisa, 2006). The interaction between MHBs and mycorrhizal fungi not only promotes the formation of mycorrhizae, but also enhances the function of the established mycorrhizal symbiosis, which in turn increases the biomass of the host plant. The role played by helper bacteria after mycorrhizal synthesis is mainly reflected in improving the level of plant mineral uptake (Jayasinghearachchi and Seneviratne, 2006; Calvaruso et al., 2007) and protecting the root system from pathogenic agents and damage (Frey-Klett and Garbaye, 2005; Nanjundappa et al., 2019). Therefore, studying colonization by MHBs is particularly important for their application in production.

In the present study, the presence of *Hymenochaete* sp. Rl significantly improved *B. pumilus* HR10 biomass of in the rhizosphere soil and on the root surface, which was consistent with the results of previous studies (Frey-Klett et al., 2007). EMC fungi form symbioses with roots, which allows the roots to not only increase their surface area to absorb mineral elements but also to expand their sphere of influence in the soil (Bais et al., 2006). EMC fungi, along with plants, are responsible for the release of various carbonaceous compounds into the soil environment, mycorrhizosphere, and microsphere, which can be used by soil bacteria, and other microorganisms as carbon and energy sources, thereby increasing the biomass of soil bacteria that can utilize these carbon and energy sources (Artursson et al., 2006; Calvaruso et al., 2007; Warmink et al., 2009). In addition, the biomass of *B. pumilus* HR10 on the root surface was significantly higher than that in the rhizosphere soil, which may be related to the quorum sensing of MHB. The surface of the roots with mycorrhizae was particularly obvious. This may be due to the increase in the number of lateral pine roots of the same length, which we simultaneously inoculated *B. pumilus* HR10 and *Hymenochaete* sp. Rl. The root surface area was increased and the biomass of *B. pumilus* HR10 was significantly increased when the roots were inoculated with *B. pumilus* HR10 and *Hymenochaete* sp. Rl together compared with that in presence of *Hymenochaete* sp. Rl only. It is also possible that the inoculation of EMC fungi can change the yield and chemical composition of the root exudates to stimulate the growth of MHB or affect the quorum sensing of MHBs to promote colonization of the mycorrhizae (Loh et al., 2002; Johansson et al., 2004; Gonzálezmula et al., 2018).

*In vitro*, neither extract had an affect the growth, spore formation, or biomass of *B. pumilus* HR10 during fermentation. The reason that *Hymenochaete* sp. Rl, *P. thunbergii* roots, and the *Hymenochaete* sp. Rl-*P. thunbergii* mycorrhizal symbiosis increase the rhizosphere biomass of *B. pumilus* HR10 may be because of their influence on its biofilm formation. Biofilm formation is an important factor affecting the colonization of MHB in the rhizosphere (Flemming et al., 2016). Bacterial strains adhere to the root surface through their self-synthesized hydrated polymers and they aggregate with other cells to form microcolonies; once the bacteria multiply and aggregate to a certain level, they begin to form the three-dimensional structure of the biofilm by secreting extracellular polysaccharides and other substances, ultimately colonizing the root surface (de Weert et al., 2004; Kohlmeier et al., 2005; Warmink and van Elsas, 2009; Miquel Guennoc et al., 2018). *Bacillus pumilus* HR10 biofilm formation and production of extracellular polysaccharides and extracellular proteins were significantly enhanced in the presence of *Hymenochaete* sp. Rl mycelium and/or *P. thunbergii* root extracts, especially when the *Hymenochaete* sp. Rl mycelium extract and the *P. thunbergii* root extracts were added together, resulting in efficient colonization of *B. pumilus* HR10 on the root surface. Because of the huge differences between experimental conditions and the soil environment, the effects of *Hymenochaete* sp. Rl, *P. thunbergii* roots and the *Hymenochaete* sp. Rl-*P. thunbergii* mycorrhizal symbiosis on the growth of *B. pumilus* HR10 in the rhizosphere environment cannot be ruled out. Numerous studies have shown that EMC fungi affect the growth of MHBs by releasing appropriate carbon sources, altering root exudates or improving the rhizosphere soil environment (Johansson et al., 2004; Toljander et al., 2006; Chen et al., 2009; Pivato et al., 2009; Meharg and Cairney, 2016; Zhou et al., 2017). The results we obtained showed that the secretions from *P. thunbergii* roots and *Hymenochaete* sp. Rl had little effect on the growth of *B. pumilus* HR10; however, the effects on the rhizosphere soil and the release of compounds after the interaction between *Hymenochaete* sp. Rl and *P. thunbergii* need further study.

We also found that *Hymenochaete* sp. Rl mycelium extracts and *P. thunbergii* root extracts can affect the chemotaxis of *B. pumilus* HR10, which might include its motility, attachment, growth and possibly swarming motility phases. The chemotactic properties are determined by the strength of bacterial flagellar motility (Lapidus et al., 1988; Pion et al., 2013; Pankratova et al., 2018). The colonization tendency and effect of root microorganisms on the root surface are related to their swimming on soft agar surfaces and swarming inside soft agars (Matthysse and Mcmahan, 2001; Esfehani et al., 2009; Hou et al., 2019). The addition of the two extracts could significantly promote the swimming of *B. pumilus* HR10 and the expression of the *fliG* gene, indicating that the addition of these extracts can improve the flagellar movement of *B. pumilus* HR10. Chemotaxis toward fungal hyphae has been observed in several studies. Kohlmeier et al. (2005) revealed that the movement of bacteria through soil, allowing them to occupy the microhabitats at the fungal hyphae, occurs by virtue of a thin water layer that surrounds the fungal hyphae. In support of the role of motility, Kohlmeier et al. (2005) observed that intrinsic motility (swimming and/or swarming) of the bacteria was required for bacterial translocation along fungal highways, as only flagellated bacterial strains could move along the hyphal surface. Toljander et al. (2006) conducted an experiment on soil bacteria tagged with green fluorescent protein to analyze the variability of bacterial attachment to AM fungal extraradical hyphae. They concluded that bacteria differ in their ability to colonize vital and nonvital hyphae and that attachment is also influenced by the fungal species involved. As bacterial motility is positively, albeit one-sided, correlated with the ability to comigrate with the growing fungal partner, a role for chemotaxis is indicated (Warmink et al., 2009; Nazir et al., 2010). Adding both extracts had no significant effect on biofilm formation or *srf* gene expression, and there was a difference between the *B. pumilus* HR10 biomass results in the rhizosphere and on the mycorrhizal surface. We hypothesize that this may be due to the interaction of *P. thunbergii*, *Hymenochaete* sp. Rl, and *B. pumilus* HR10 in the rhizosphere and that the interaction between *Hymenochaete* sp. Rl and *P. thunbergii* alters the yield or chemical composition of the root exudates of *P. thunbergii*, thus causing different impacts on the chemotaxis of *B. pumilus* HR10.

In conclusion, *B. pumilus* HR10 and *Hymenochaete* sp. Rl inoculation increased the biomass of *B. pumilus* HR10 in the rhizosphere soil and on the root surface compared with *B. pumilus* HR10 inoculation solely, while extracts of *P. thunbergii* and *Hymenochaete* sp. Rl enhanced biofilm formation and the expression of colonization-related genes in *B. pumilus* HR10, indicating that the ectomycorrhizal mycorrhizal fungus *Hymenochaete* sp. Rl-*P. thunbergii* mycorrhizal symbiosis could promote the survival and colonization of *B. pumilus* HR10 in the rhizosphere. This study provides support for the tripartite interactions of MHBs, mycorrhizal fungi and host plants.

## Data Availability Statement

The original contributions presented in the study are included in the article/Supplementary Material, further inquiries can be directed to the corresponding author.

## Author Contributions

X-QW and Y-HW conceptualized the idea for the study. Y-HW performed most of experimental operations and data analysis and led the writing of the manuscript. X-QW critically reviewed the data analysis and contributed substantially to the writing. W-LK, M-LZ, and YD made key suggestions for improving the paper. All authors have participated in the preparation of this study. All authors contributed to the article and approved the submitted version.

## Funding

This work was supported by the National Key Research and Development Program of China (2017YFD0600104) and the Priority Academic Program Development of Jiangsu Higher Education Institutions (PAPD).

## Conflict of Interest

The authors declare that the research was conducted in the absence of any commercial or financial relationships that could be construed as a potential conflict of interest.

## Publisher’s Note

All claims expressed in this article are solely those of the authors and do not necessarily represent those of their affiliated organizations, or those of the publisher, the editors and the reviewers. Any product that may be evaluated in this article, or claim that may be made by its manufacturer, is not guaranteed or endorsed by the publisher.

## References

[ref1] Aarle I. Söderström B. Olsson P. A. (2003). Growth and interactions of arbuscular mycorrhizal fungi in soils from limestone and acid rock habitats. Soil Biol. Biochem. 35, 1557–1564. doi: 10.1016/S0038-0717(03)00248-7

[ref2] Albertsen A. Ravnskov S. Green H. Jensen D. F. Larsen J. (2006). Interactions between the external mycelium of the mycorrhizal fungus *Glomus intraradices* and other soil microorganisms as affected by organic matter. Soil Biol. Biochem. 38, 1008–1014. doi: 10.1016/j.soilbio.2005.08.015

[ref3] Armada E. Probanza A. Roldan A. Azcon R. (2016). Native plant growth promoting bacteria *Bacillus thuringiensis* and mixed or individual mycorrhizal species improved drought tolerance and oxidative metabolism in *Lavandula dentata* plants. J. Plant Physiol. 192, 1–12. doi: 10.1016/j.jplph.2015.11.007, PMID: 26796423

[ref4] Artursson V. Finlay R. D. Jansson J. K. (2006). Interactions between arbuscular mycorrhizal fungi and bacteria and their potential for stimulating plant growth. Environ. Microbiol. 8, 1–10. doi: 10.1111/j.1462-2920.2005.00942.x, PMID: 16343316

[ref5] Aspray T. J. Frey-Klett P. Jones J. E. Whipps J. M. Garbaye J. Bending G. D. (2006). Mycorrhization helper bacteria: a case of specificity for altering ectomycorrhiza architecture but not ectomycorrhiza formation. Mycorrhiza 16, 533–541. doi: 10.1007/s00572-006-0068-3, PMID: 16983568

[ref6] Bais H. P. Weir T. L. Perry L. G. Gilroy S. Vivanco J. M. (2006). The role of root exudates in rhizosphere interations with plants and other organisms. Annu. Rev. Plant Biol. 57, 233–266. doi: 10.1146/annurev.arplant.57.032905.105159, PMID: 16669762

[ref7] Calvaruso C. Turpault M.-P. Leclerc E. Frey-Klett P. (2007). Impact of ectomycorrhizosphere on the functional diversity of soil bacterial and fungal communities from a forest stand in relation to nutrient mobilization processes. Microb. Ecol. 54, 567–577. doi: 10.1007/s00248-007-9260-z, PMID: 17546519

[ref8] Cavagnaro T. R. Jackson L. E. Six J. Ferris H. Goyal S. Asami D.. (2006). Arbuscular mycorrhizas, microbial communities, nutrient availability, and soil aggregates in organic tomato production. Plant Soil 282, 209–225. doi: 10.1007/s11104-005-5847-7

[ref9] Chen M.-M. Chen B.-D. Xu Y. Tian H.-Y. Deng H. (2009). Mycorrhizal fungi in bioremediation of petroleum-contaminated soil: a review. Chin. J. Ecol. 28, 1171–1177.

[ref10] Christensen H. Jakobsen I. (1993). Reduction of bacterial growth by a vesicular-arbuscular mycorrhizal fungus in the rhizosphere of cucumber (*Cucumis sativus* L.). Biol. Fertil. Soils 15, 253–258. doi: 10.1007/BF00337209

[ref11] Dai Y. Wu X.-Q. Wang Y.-H. Zhu M.-L. (2021). Biocontrol potential of *Bacillus pumilus* HR10 against *Sphaeropsis* shoot blight disease of pine. Biol. Control 152:104458. doi: 10.1016/j.biocontrol.2020.104458

[ref13] Deveau A. Brule C. Palin B. Champmartin D. Rubini P. Garbaye J.. (2010). Role of fungal trehalose and bacterial thiamine in the improved survival and growth of the ectomycorrhizal fungus *Laccaria bicolor* S238N and the helper bacterium *Pseudomonas fluorescens* BBc6R8. Environ. Microbiol. Rep. 2, 560–568. doi: 10.1111/j.1758-2229.2010.00145.x, PMID: 23766226

[ref14] Deveau A. Palin B. Delaruelle C. Peter M. Kohler A. Pierrat J. C.. (2007). The mycorrhiza helper *Pseudomonas fluorescens* BBc6R8 has a specific priming effect on the growth, morphology and gene expression of the ectomycorrhizal fungus *Laccaria bicolor* S238N. New Phytol. 175, 743–755. doi: 10.1111/j.1469-8137.2007.02148.x, PMID: 17688589

[ref12] De Weert S. Kuiper I. Lagendijk E. L. Lamers G. E. M. Lugtenberg B. J. J. (2004). Role of chemotaxis toward fusaric acid in colonization of hyphae of *Fusarium oxysporum* f. sp radicis-lycopersici by *Pseudomonas fluorescens* WCS365. Mol. Plant-Microbe Interact. 17, 1185–1191. doi: 10.1094/MPMI.2004.17.11.1185, PMID: 15553244

[ref15] Dietz S. Von Bulow J. Beitz E. Nehls U. (2011). The aquaporin gene family of the ectomycorrhizal fungus *Laccaria bicolor*: lessons for symbiotic functions. New Phytol. 190, 927–940. doi: 10.1111/j.1469-8137.2011.03651.x, PMID: 21352231

[ref16] Duponnois R. Kisa M. (2006). The possible role of trehalose in the mycorrhiza helper bacterium effect. Can. J. Bot. 84, 1005–1008. doi: 10.1139/b06-053

[ref17] Esfehani Y. J. Khavazi K. Ghorbani S. (2009). Cross interaction of *Pseudomonas putida* and *Glomus intraradices* and its effect on wheat root colonization. Pak. J. Biol. Sci. 12, 1365–1370. doi: 10.3923/pjbs.2009.1365.1370, PMID: 20128504

[ref18] Flemming H. C. Wingender J. Szewzyk U. Steinberg P. Rice S. A. Kjelleberg S. (2016). Biofilms: an emergent form of bacterial life. Nat. Rev. Microbiol. 14, 563–575. doi: 10.1038/nrmicro.2016.94, PMID: 27510863

[ref19] Frey-Klett P. Chavatte M. Clausse M. L. Courrier S. Le Roux C. Raaijmakers J.. (2005). Ectomycorrhizal symbiosis affects functional diversity of rhizosphere *fluorescent pseudomonads*. New Phytol. 165, 317–328. doi: 10.1111/j.1469-8137.2004.01212.x, PMID: 15720643

[ref20] Frey-Klett P. Garbaye J. (2005). Mycorrhiza helper bacteria: a promising model for the genomic analysis of fungal-bacterial interactions. New Phytol. 168, 4–8. doi: 10.1111/j.1469-8137.2005.01553.x, PMID: 16159316

[ref21] Frey-Klett P. Garbaye J. Tarkka M. (2007). The mycorrhiza helper bacteria revisited. New Phytol. 176, 22–36. doi: 10.1111/j.1469-8137.2007.02191.x, PMID: 17803639

[ref22] Frey-Klett P. Pierrat J. C. Garbaye J. (1997). Location and survival of mycorrhiza helper *Pseudomonas fluorescens* during establishment of ectomycorrhizal symbiosis between *Laccaria bicolor* and *Douglas fir*. Appl. Environ. Microbiol. 63, 139–144. doi: 10.1128/aem.63.1.139-144.1997, PMID: 16535478 PMC1389093

[ref23] Gamalero E. Lingua G. Tombolini R. Avidano L. Pivato B. Berta G. (2005). Colonization of tomato root seedling by *Pseudomonas fluorescens* 92rkG5: spatio-temporal dynamics, localization, organization, viability, and culturability. Microb. Ecol. 50, 289–297. doi: 10.1007/s00248-004-0149-9, PMID: 16211326

[ref24] Gamalero E. Trotta A. Massa N. Copetta A. Martinotti M. G. Berta G. (2004). Impact of two fluorescent pseudomonads and an arbuscular mycorrhizal fungus on tomato plant growth, root architecture and P acquisition. Mycorrhiza 14, 185–192. doi: 10.1007/s00572-003-0256-3, PMID: 15197635

[ref25] Garbaye J. (1994). Helper bacteria—a new dimension to the mycorrhizal symbiosis. New Phytol. 128, 197–210. doi: 10.1111/j.1469-8137.1994.tb04003.x, PMID: 33874371

[ref26] Gonzálezmula A. Lang J. Grandclément C. Naquin D. Ahmar M. Soulère L.. (2018). Lifestyle of the biotroph *Agrobacterium tumefaciens* in the ecological niche constructed on its host plant. New Phytol. 219, 350–362. doi: 10.1111/nph.15164, PMID: 29701262

[ref27] Guennoc C. M. Rose C. Labbe J. Deveau A. (2018). Bacterial biofilm formation on the hyphae of ectomycorrhizal fungi: a widespread ability under controls? FEMS Microbiol. Ecol. 94:fiy093. doi: 10.1093/femsec/fiy093, PMID: 29788056

[ref28] Guo X. Chen H. Y. H. Meng M. Biswas S. R. Ye L. Zhang J. (2016). Effects of land use change on the composition of soil microbial communities in a managed subtropical forest. For. Ecol. Manag. 373, 93–99. doi: 10.1016/j.foreco.2016.03.048

[ref29] Hou L. Debru A. Chen Q. Bao Q. Li K. (2019). AmrZ regulates swarming motility through cyclic di-GMP-dependent motility inhibition and controlling Pel polysaccharide production in *Pseudomonas aeruginosa* PA14. Front. Microbiol. 10:1847. doi: 10.3389/fmicb.2019.01847, PMID: 31474950 PMC6707383

[ref30] Jayasinghearachchi H. S. Seneviratne G. (2006). Fungal solubilization of rock phosphate is enhanced by forming fungal-rhizobial biofilms. Soil Biol. Biochem. 38, 405–408. doi: 10.1016/j.soilbio.2005.06.004

[ref31] Johansson J. F. Paul L. R. Finlay R. D. (2004). Microbial interactions in the mycorrhizosphere and their significance for sustainable agriculture. FEMS Microbiol. Ecol. 48, 1–13. doi: 10.1016/j.femsec.2003.11.012, PMID: 19712426

[ref32] Kohlmeier S. Smits T. H. M. Ford R. M. Keel C. Harms H. Wick L. Y. (2005). Taking the fungal highway: mobilization of pollutant-degrading bacteria by fungi. Environ. Sci. Technol. 39, 4640–4646. doi: 10.1021/es047979z, PMID: 16047804

[ref33] Labbe J. L. Weston D. J. Dunkirk N. Pelletier D. A. Tuskan G. A. (2014). Newly identified helper bacteria stimulate ectomycorrhizal formation in *Populus*. Front. Plant Sci. 5:579. doi: 10.3389/fpls.2014.00579, PMID: 25386184 PMC4208408

[ref34] Lapidus I. R. Welch M. Eisenbach M. (1988). Pausing of flagellar rotation is a component of bacterial motility and chemotaxis. J. Bacteriol. 170, 3627–3632. doi: 10.1128/jb.170.8.3627-3632.1988, PMID: 3042756 PMC211337

[ref35] Li Y. Wang S. Lu M. Zhang Z. Chen M. Li S.. (2019). Rhizosphere interactions between earthworms and arbuscular mycorrhizal fungi increase nutrient availability and plant growth in the desertification soils. Soil Tillage Res. 186, 146–151. doi: 10.1016/j.still.2018.10.016

[ref36] Loh J. Pierson E. A. Iii L. S. P. Stacey G. Chatterjee A. (2002). Quorum sensing in plant-associated bacteria. Curr. Opin. Plant Biol. 5, 285–290. doi: 10.1016/S1369-5266(02)00274-1, PMID: 12179960

[ref37] Martin F. Kohler A. Murat C. Veneault-Fourrey C. Hibbett D. S. (2016). Unearthing the roots of ectomycorrhizal symbioses. Nat. Rev. Microbiol. 14, 760–773. doi: 10.1038/nrmicro.2016.149, PMID: 27795567

[ref38] Marupakula S. Mahmood S. Finlay R. D. (2016). Analysis of single root tip microbiomes suggests that distinctive bacterial communities are selected by *Pinus sylvestris* roots colonized by different ectomycorrhizal fungi. Environ. Microbiol. 18, 1470–1483. doi: 10.1111/1462-2920.13102, PMID: 26521936

[ref39] Matthysse A. G. Mcmahan S. (2001). The effect of the *Agrobacterium tumefaciens* attR mutation on attachment and root colonization differs between legumes and other dicots. Appl. Environ. Microbiol. 67, 1070–1075. doi: 10.1128/AEM.67.3.1070-1075.2001, PMID: 11229893 PMC92696

[ref40] Mechri B. Manga A. G. B. Tekaya M. Attia F. Cheheb H. Ben Meriem F.. (2014). Changes in microbial communities and carbohydrate profiles induced by the mycorrhizal fungus (*Glomus intraradices*) in rhizosphere of olive trees (*Olea europaea* L.). Appl. Soil Ecol. 75, 124–133. doi: 10.1016/j.apsoil.2013.11.001

[ref41] Meharg A. A. Cairney J. W. G. (2016). Ectomycorrhizas—extending the capabilities of rhizosphere remediation? Soil Biol. Biochem. 32, 1475–1484. doi: 10.1016/S0038-0717(00)00076-6

[ref42] Moreau D. Bardgett R. D. Finlay R. D. Jones D. L. Philippot L. (2019). A plant perspective on nitrogen cycling in the rhizosphere. Funct. Ecol. 33, 540–552. doi: 10.1111/1365-2435.13303

[ref43] Morgado L. N. Semenova T. A. Welker J. M. Walker M. D. Smets E. Geml J. (2015). Summer temperature increase has distinct effects on the ectomycorrhizal fungal communities of moist tussock and dry tundra in Arctic Alaska. Glob. Chang. Biol. 21, 959–972. doi: 10.1111/gcb.12716, PMID: 25156129 PMC4322476

[ref44] Nanjundappa A. Bagyaraj D. J. Na A. K. Kumar M. Chakdar H. (2019). Interaction between arbuscular mycorrhizal fungi and *Bacillus* spp. in soil enhancing growth of crop plants. Fungal Biol. Biotechnol. 6:23. doi: 10.1186/s40694-019-0086-5, PMID: 31798924 PMC6882151

[ref45] Nazir R. Warmink J. A. Boersma H. Van Elsas J. D. (2010). Mechanisms that promote bacterial fitness in fungal-affected soil microhabitats. FEMS Microbiol. Ecol. 71, 169–185. doi: 10.1111/j.1574-6941.2009.00807.x, PMID: 20002182

[ref46] Oldroyd G. E. D. Leyser O. (2020). A plant's diet, surviving in a variable nutrient environment. Science 368:eaba0196. doi: 10.1126/science.aba0196, PMID: 32241923

[ref47] Olsson P. A. Bååth E. Jakobsen I. Söderström B. (1996). Soil bacteria respond to presence of roots but not to mycelium of arbuscular mycorrhizal fungi. Soil Biol. Biochem. 28, 463–470. doi: 10.1016/0038-0717(96)00011-9

[ref48] Pankratova E. V. Kalyakulina A. I. Krivonosov M. I. Denisov S. V. Taute K. M. Zaburdaev V. Y. (2018). Chemotactic drift speed for bacterial motility pattern with two alternating turning events. PLoS One 13:e0190434. doi: 10.1371/journal.pone.0190434, PMID: 29351336 PMC5774696

[ref49] Pion M. Bshary R. Bindschedler S. Filippidou S. Wick L. Y. Job D.. (2013). Gains of bacterial flagellar motility in a fungal world. Appl. Environ. Microbiol. 79, 6862–6867. doi: 10.1128/AEM.01393-13, PMID: 23995942 PMC3811526

[ref50] Pivato B. Offre P. Marchelli S. Barbonaglia B. Mougel C. Lemanceau P.. (2009). Bacterial effects on arbuscular mycorrhizal fungi and mycorrhiza development as influenced by the bacteria, fungi, and host plant. Mycorrhiza 19, 81–90. doi: 10.1007/s00572-008-0205-2, PMID: 18941805

[ref51] Plassard C. Dell B. (2010). Phosphorus nutrition of mycorrhizal trees. Tree Physiol. 30, 1129–1139. doi: 10.1093/treephys/tpq063, PMID: 20631011

[ref52] Poole E. J. Bending G. D. Whipps J. M. Read D. J. (2001). Bacteria associated with *Pinus sylvestri*-*Lactarius rufus* ectomycorrhizas and their effects on mycorrhiza formation in vitro. New Phytol. 151, 743–751. doi: 10.1046/j.0028-646x.2001.00219.x, PMID: 33853249

[ref53] Santalahti M. Sun H. Sietio O.-M. Koster K. Berninger F. Laurila T.. (2018). Reindeer grazing alter soil fungal community structure and litter decomposition related enzyme activities in boreal coniferous forests in Finnish Lapland. Appl. Soil Ecol. 132, 74–82. doi: 10.1016/j.apsoil.2018.08.013

[ref54] Schrey S. D. Schellhammer M. Ecke M. Hampp R. Tarkka M. T. (2005). Mycorrhiza helper bacterium *Streptomyces* AcH 505 induces differential gene expression in the ectomycorrhizal fungus *Amanita muscaria*. New Phytol. 168, 205–216. doi: 10.1111/j.1469-8137.2005.01518.x, PMID: 16159334

[ref55] Sheng J.-M. Wu X.-Q. Hou L.-L. (2014). Isolating and identifying mycorrhiza helper bacteria from the rhizosphere soil of *Pinus thunbergi* inoculated with *Rhizipogen luteous*. Chin. J. North. For. Univ. 42, 110–114. doi: 10.13759/j.cnki.dlxb.20140522.036

[ref56] Shinde S. Zerbs S. Collart F. R. Cumming J. R. Noirot P. Larsen P. E. (2019). *Pseudomonas fluorescens* increases mycorrhization and modulates expression of antifungal defense response genes in roots of aspen seedlings. BMC Plant Biol. 19:13. doi: 10.1186/s12870-018-1610-0, PMID: 30606121 PMC6318872

[ref57] Steidinger B. S. Crowther T. W. Liang J. Van Nuland M. E. Werner G. D. A. Reich P. B.. (2019). Climatic controls of decomposition drive the global biogeography of forest-tree symbioses. Nature 569, 404–408. doi: 10.1038/s41586-019-1128-0, PMID: 31092941

[ref58] Tedersoo L. Bahram M. Zobel M. (2020). How mycorrhizal associations drive plant population and community biology. Science 367:eaba1223. doi: 10.1126/science.aba1223, PMID: 32079744

[ref59] Toljander J. F. Artursson V. Paul L. R. Jansson J. K. Finlay R. D. (2006). Attachment of different soil bacteria to arbuscular mycorrhizal fungal extraradical hyphae is determined by hyphal vitality and fungal species. FEMS Microbiol. Lett. 254, 34–40. doi: 10.1111/j.1574-6968.2005.00003.x, PMID: 16451176

[ref60] Velez P. Espinosa-Asuar L. Figueroa M. Gasca-Pineda J. Aguirre-Von-Wobeser E. Eguiarte L. E.. (2018). Nutrient dependent cross-kingdom interactions: fungi and bacteria from an oligotrophic desert oasis. Front. Microbiol. 9:1755. doi: 10.3389/fmicb.2018.01755, PMID: 30131780 PMC6090137

[ref61] Vivas A. Biro B. Ruiz-Lozano J. M. Barea J. M. Azcon R. (2006). Two bacterial strains isolated from a Zn-polluted soil enhance plant growth and mycorrhizal efficiency under Zn-toxicity. Chemosphere 62, 1523–1533. doi: 10.1016/j.chemosphere.2005.06.053, PMID: 16098559

[ref62] Wamberg C. Christensen S. Jakobsen I. Müller A. Rensen S. J. (2003). The mycorrhizal fungus (*Glomus intraradices*) affects microbial activity in the rhizosphere of pea plants (*Pisum sativum*). Soil Biol. Biochem. 35, 1349–1357. doi: 10.1016/S0038-0717(03)00214-1

[ref63] Wang Y.-H. Hou L.-L. Wu X.-Q. Zhu M.-L. Dai Y. Zhao Y.-J. (2021). Mycorrhiza helper bacterium *Bacillus pumilus* HR10 the improves growth and nutritional status of *Pinus thunbergii* by promoting mycorrhizal proliferation. Tree Physiol. 3:tpab139. doi: 10.1093/treephys/tpab139, PMID: 34730183

[ref64] Warmink J. A. Nazir R. Van Elsas J. D. (2009). Universal and species-specific bacterial 'fungiphiles' in the mycospheres of different basidiomycetous fungi. Environ. Microbiol. 11, 300–312. doi: 10.1111/j.1462-2920.2008.01767.x, PMID: 19196267

[ref65] Warmink J. A. Van Elsas J. D. (2009). Migratory response of soil bacteria to *Lyophyllum* sp. strain Karsten in soil microcosms. Appl. Environ. Microbiol. 75, 2820–2830. doi: 10.1128/AEM.02110-08, PMID: 19286795 PMC2681705

[ref66] Yang X.-M. (2004). The Collection and Application Studies on the Mycorrhiza Fungi of Pinus Tree. Nanjing, China: Nanjing Forestry University.

[ref67] Zhao W. Li Y. Gao P. Sun Z. Sun T. Zhang H. (2011). Validation of reference genes for real-time quantitative PCR studies in gene expression levels of *Lactobacillus casei* Zhang. J. Ind. Microbiol. Biotechnol. 38, 1279–1286. doi: 10.1007/s10295-010-0906-3, PMID: 21104423

[ref68] Zhao L. Wu X.-Q. Ye J.-R. Li H. Li G.-E. (2013). Isolation and characterization of a mycorrhiza helper bacterium from rhizosphere soils of poplar stands. Biol. Fertil. Soils 50, 593–601. doi: 10.1007/s00374-013-0880-9

[ref69] Zhou Q. Mu K. Xu M. Ma X. Ni Z. Wang J.. (2017). Variation in the concentrations of major secondary metabolites in ginkgo leaves from different geographical populations. Forests 8:266. doi: 10.3390/f8080266

[ref70] Zhu Y. L. Hou H. M. Zhang G. L. Wang Y. F. Hao H. S. (2019). AHLs regulate biofilm formation and swimming motility of *Hafnia alvei* H4. Front. Microbiol. 10:1330. doi: 10.3389/fmicb.2019.01330, PMID: 31275267 PMC6593095

[ref71] Zhu M. L. Wu X. Q. Wang Y. H. Dai Y. (2020). Role of biofilm formation by *Bacillus pumilus* HR10 in biocontrol against pine seedling damping-off disease caused by *Rhizoctonia solani*. Forests 11:652. doi: 10.3390/f11060652

